# Cancer patients' attitudes towards Chinese medicine: a Hong Kong survey

**DOI:** 10.1186/1749-8546-4-25

**Published:** 2009-12-30

**Authors:** Yuen-chi Lam, Chung-wah Cheng, Heng Peng, Chun-key Law, Xianzhang Huang, Zhaoxiang Bian

**Affiliations:** 1 School of Chinese Medicine, Hong Kong Baptist University, Hong Kong SAR, China; 2 Department of Mathematics, Hong Kong Baptist University, Hong Kong SAR, China; 3 Department of Clinical Oncology, Queen Elizabeth Hospital, Hong Kong SAR, China

## Abstract

**Background:**

This article reports a survey conducted in Hong Kong on the cancer patients' attitudes towards Chinese medicine treatment.

**Methods:**

Cancer patients from three Chinese medicine clinics and one oncology clinic were interviewed with a structured questionnaire.

**Results:**

Of a total of 786 participants included in the study, 42.9% used Western medicine only; 57.1% used at least one form of Chinese medicine; 5 participants used Chinese medicine only; and 56.5% used Chinese medicine before/during/after Western medicine treatment. Commonly used Western medicine and Chinese medicine treatments included chemotherapy (63.7%), radiotherapy (62.0%), surgery (57.6%), Chinese herbal medicine (53.9%) and Chinese dietary therapy (9.5%). Participants receiving chemotherapy used Chinese medicine (63.3%) more than those receiving any other Western medicine treatments. Spearman correlation coefficients showed that the selection of Chinese medicine was associated with the cancer type (r_s _= -1.36; *P *< 0.001), stage (r_s _= 0.178; *P *< 0.001), duration (r_s _= -0.074; *P *= 0.037), whether receiving chemotherapy (r_s _= 0.165; *P *< 0.001) and palliative therapy (r_s _= 0.087; *P *= 0.015). Nearly two-thirds of the participants (N = 274) did not tell their physicians about using Chinese medicine. Over two-thirds of all participants (68.2%) believed that integrated Chinese and Western medicine was effective.

**Conclusion:**

Chinese medicine is commonly used among Hong Kong cancer patients. The interviewed cancer patients in Hong Kong considered integrative Chinese and Western medicine is an effective cancer treatment.

## Background

Cancer is a major disease in Hong Kong with great social and economic burden. According to the Hong Kong Cancer Registry, 23,750 new cancer cases and 12,093 cancer deaths were recorded in 2006. New cancer cases in Hong Kong has been rising at a annual rate of 2% [1]. While surgery, radiotherapy and chemotherapy remained to be conventional cancer treatments, 80% of the cancer patients around the world consult complementary and alternative medicine (CAM) for more treatment options [2-5]. Chinese medicine, one of the most popular CAMs, is an available option in many cancer centres in Asia [6-8], North America [9,10], and Europe [5].

Chinese medicine and Western medicine differ fundamentally in their etiological concepts and therapeutic approaches. In Western medicine, cancer is perceived as uncontrolled growth of malignant cells which may be treated by surgery, chemotherapy, and radiotherapy [11]. According to Chinese medicine theory, cancer is the manifestation of a *qi *disturbance which may be treated by mobilizing *qi*. Study results support the use of Chinese medicine to treat liver cancer and leukaemia [12,13], and recent meta-analyses demonstrated that Chinese medicine improved tumor response to chemotherapy as well as patient's survival rates [14,15]. Five common Chinese medicine modalities, namely Chinese herbal medicine, acupuncture and moxibustion, therapeutic massage, *qigong *and Chinese dietary therapy have been used to treat cancer [16]. Moreover, acupuncture relieves pain and acute vomiting during conventional cancer treatment [15,17]

While some researchers suggest that Chinese medicine should be integrated into a comprehensive cancer treatment scheme [18], cancer patients' attitude towards Chinese medicine is largely unknown. The present study aimed to reveal the prevalence and pattern of the use of Chinese medicine among cancer patients in Hong Kong and to assess their attitudes and intentions about such use.

## Methods

### Participants

This study was approved by the Committee on the Use of Human and Animal Subjects in Teaching and Research of the Hong Kong Baptist University (HKBU) and the Research Ethics Committee of the Hospital Authority (HA) Hong Kong. Between April 2008 and August 2008, all cancer patients attending any of the three HKBU Chinese medicine clinics and the outpatient clinics of the Department of Clinical Oncology in Queen Elizabeth Hospital (QEH) were invited to participate in this cross-sectional survey.

Oral informed consent was obtained from cancer patients before participation. Each participant completed a questionnaire, which was then checked by one of the authors (YCL). Completed and checked questionnaires were coded to mask patients' identities. Another author (CWC) double-checked the collected questionnaire to ensure good quality.

In this paper, Chinese medicine user is defined as the person who receives treatments of Chinese herbal medicine, acupuncture and moxibustion, therapeutic massage, *qigong*, Chinese dietary therapy and/or other therapies that are based on the theory of Chinese medicine.

### Questionnaire

The development of the questionnaire (in Chinese language) included four stages as follows (1) a draft questionnaire was prepared; (2) the questionnaire was reviewed by Chinese medicine experts (N = 6) and Western medicine practitioners (N = 2) were collected; (3) the draft questionnaire was revised by the authors and tested on a small group (N = 10) of cancer patients;.(4) the questionnaire was finalized. The final questionnaire consisted of three parts. The first part was about background information of the participants (e.g. age, gender, marital status, educational level), type of cancer, date of diagnosis, use of Western medicine cancer treatment and/or Chinese medicine treatment. The second part focused on participants' perception of Western medicine treatment and/or Chinese medicine treatment, such as times to initiate Chinese medicine treatment, motivations for using Chinese medicine, whether or not their physicians were told about the use of Chinese medicine and why, responses from physicians, reasons for choosing Western medicine treatment and/or Chinese medicine treatment, satisfaction with the treatment. Finally, participants were asked whether they intended to continue or stop their use of Western medicine treatment and/or Chinese medicine treatment, or other CAM therapies in the future, and their opinion on the effectiveness of the integration of Chinese and Western medicine in their cancer treatment. In the third part, participants were asked to express their views on the integration of Chinese medicine and Western medicine in cancer treatment.

### Data management and statistical analyses

YCL, one of the authors, entered the data into an Access (Microsoft, USA) database, and CWC, another author, checked the data independently. All data disagreements were resolved through further checks against raw data. Data were analyzed with the Statistical Package for Social Sciences program (SPSS 13.0, SPSS, USA). Demographic and clinical characteristic differences between Western medicine and Chinese medicine users were assessed with chi-square test. Spearman correlation between the use of Chinese medicine and other variables of interest was determined. All statistical tests were two-tailed with a confidence level of alpha of 0.05.

## Results

### Demographic and clinical characteristics of study participants

Seven hundred and ninety-one (791) questionnaires were distributed, of which 786 (99.4%) received responses. Common cancers among the participants were lung cancer, breast cancer, colorectal cancer and nasopharyngeal cancer (Table 1).

**Table 1:** Demographic characteristics of the study population

Variables	No. of patients n(%)	Western medicine n(%)	Chinese medicine n(%)	*P*-value
Total	786(100.0)	337(42.9)	449(57.1)	
Age, years				0.061
≦40	92(11.7)	45(13.4)	47(10.5)	
41-50	218(27.7)	78(23.1)	140(31.2)	
51-60	219(27.9)	94(27.9)	125(27.8)	
≧61	257(32.7)	120(35.6)	135(30.5)	
Gender				0.285
Male	339(43.1)	138(40.9)	201(44.8)	
Female	447(56.9)	199(59.1)	248(55.2)	
Clinical setting				< 0.001
Chinese medicine Clinic	117(14.9)	2(0.6)	115(25.6)	
Western medicine Clinic	669(85.1)	335(99.4)	334(74.4)	
Marital status				0.200
Married	659(83.8)	276(81.9)	383(85.3)	
Unmarried	127(16.2)	61(18.1)	66(14.7)	
Education level				0.001
Elementary school	244(31.0)	120(35.6)	124(27.6)	
High school	374(47.6)	155(46.0)	219(48.8)	
College or University	114(14.5)	33(9.8)	81(18.0)	
Other	54(6.9)	29(8.6)	25(5.6)	

Data are presented as the actual number of patients (percentage in that group). Chi-square tests were conducted between Western medicine and Chinese medicine users.

Compared with Western medicine users, Chinese medicine users were better educated among whom Stage III or IV lung cancer, breast cancer and nasopharyngeal cancer and cancers which had been diagnosed within the last 36 months (*P *< 0.05).

### Prevalence and pattern of treatment

Nearly all participants (99.4%) used western medicine; 56.5% (N = 444) combined Western medicine with Chinese medicine, while 42.9% (N = 337) used Western medicine alone. Only 0.6% (N = 5) of the participants used Chinese medicine alone. This pattern was probably skewed towards Western medicine users because 85.1% (N = 669) participants were recruited from Western medicine clinics. Nevertheless, almost half of those patients received Chinese medicine treatment. Furthermore, the Spearman correlation coefficient study showed type of cancer (r_s _= -1.36; *P *< 0.001), stage of cancer (r_s _= 0.178; *P *< 0.001), time since diagnosis (r_s _= -0.074; *P *= 0.037) were correlated with the use of Chinese medicine (Tables 1, 2, 3).

**Table 2:** Clinical characteristics of study population

Variables	No. of patients n(%)	Western medicine n(%)	Chinese medicine n(%)	*P*-value
Total	786(100.0)	337(42.9)	449(57.1)	
Cancer type				< 0.001
Lung	145(18.4)	46(13.6)	99(22.0)	
Breast	128(16.3)	50(14.8)	78(17.4)	
Prostate	22(2.8)	11(3.3)	11(2.4)	
Colorectal	114(14.5)	53(15.7)	61(13.6)	
Liver	13(1.7)	1(0.3)	12(2.7)	
Stomach	15(1.9)	4(1.2)	11(2.4)	
Nasopharyngeal	117(14.9)	45(13.4)	72(16.0)	
Cervical	17(2.2)	12(3.6)	5(1.1)	
Endometrial	23(2.9)	13(3.9)	10(2.2)	
Ovary	18(2.3)	12(3.6)	6(1.3)	
Other	174(22.1)	90(26.7)	84(18.7)	
				
Stage (TNM)				< 0.001
0-I	106(13.5)	56(16.6)	50(11.1)	
II	122(15.5)	49(14.5)	73(16.3)	
III	202(25.7)	71(21.1)	131(29.2)	
IV	116(14.8)	35(10.4)	81(18.0)	
Unknown	240(30.5)	126(37.5)	114(25.4)	
				
Time since diagnosis (months)				0.005
≦36	537(68.3)	216(64.1)	321(71.5)	
>36, ≦48	59(7.5)	23(6.8)	36(8.0)	
>48, <60	27(3.4)	9(2.7)	18(4.0)	
≧60	153(19.5)	81(24.0)	72(16.0)	
Unknown	10(1.3)	8(2.4)	2(0.4)	

Data are presented as the actual number of patients (percentage in that group). Chi-square tests were conducted between Western medicine and Chinese medicine users.

**Table 3:** Prevalence and patterns of treatments

Variables	No. of patients n(%)	Western medicine n(%)	Chinese medicine n(%)	*P*-value
Total	786(100)	337(42.9)	449(57.1)	
Conventional treatment	781(99.4)	337(100)	444(98.9)	0.052
Surgery	453(57.6)	196(58.2)	257(57.2)	0.796
Chemotherapy	501(63.7)	184(54.6)	317(70.6)	< 0.001
Radiotherapy	487(62.0)	208(61.7)	279(62.1)	0.905
Endocrine therapy	84(10.7)	36(10.7)	48(10.7)	0.997
Palliative therapy	12(1.5)	1(0.3)	11(2.4)	0.015

Chinese medicine	449(57.1)	---	449(100)	---
treatment	424(53.9)	---	424(94.4)	
Chinese Herbal Medicine	21(2.7)	---	21(4.7)	
Acupuncture and Moxibustion	11(1.4)	---	11(2.4)	
Therapeutic Massage	40(5.1)	---	40(8.9)	
*Qigong*	75(9.5)	---	75(16.7)	
Chinese Dietary Therapy				

Data are presented as the actual number of patients (percentage in that group) who received Western medicine and Chinese medicine treatments.

**Figure 1:**
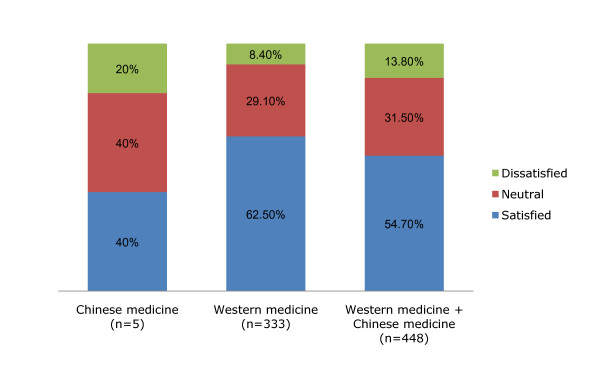
**Summary of factors contributing to patients' preferences towards cancer treatments**.

The use profile of Western medicine treatment among the participants was as follows: chemotherapy (63.7%), radiotherapy (62.0%), surgery (57.6%), endocrine therapy (10.7%), and palliative therapy (1.5%) (Table 4). Chinese herbal medicine (94.4%) was the most used Chinese medicine modality, followed by Chinese dietary therapy (16.7%) *qigong *(8.9%), acupuncture and moxibustion (4.7%) and therapeutic massage (2.4%) (Table 3). Results showed that 62.6% of the participants received only Western medicine and 54.3% of the participants (N = 244) received both Western medicine and Chinese medicine were satisfied with their treatments (Figure 1).

**Table 4:** Integrated pattern between Chinese treatment and five common anti-cancer Western medicine treatments

Types of Chinese medicine treatment	No. of users	No. of Chinese medicine users (%)	Types of Chinese medicine treatment	No. of users (%)
Total	781	449(57.1)		

Surgery	453	257(56.7)	Chinese Herbal Medicine	243(53.6)
			Acupuncture and Moxibustion	8(1.8)
			Therapeutic Massage	4(0.9)
			*Qigong*	25(5.5)
			Chinese Dietary Therapy	51(11.3)

Chemotherapy	501	317(63.3)	Chinese Herbal Medicine	301(60.1)
			Acupuncture and Moxibustion	12(2.4)
			Therapeutic Massage	6(1.2)
			*Qigong*	28(5.6)
			Chinese Dietary Therapy	50(10.0)

Radiotherapy	487	279(57.3)	Chinese Herbal Medicine	265(54.4)
			Acupuncture and Moxibustion	14(2.9)
			Therapeutic Massage	8(1.6)
			*Qigong*	24(4.9)
			Chinese Dietary Therapy	49(10.1)

Endocrine therapy	84	48(57.1)	Chinese Herbal Medicine	47(56.0)
			Acupuncture and Moxibustion	2(2.4)
			Therapeutic Massage	2(2.4)
			*Qigong*	8(9.5)
			Chinese Dietary Therapy	11(13.1)

Palliative therapy	12	11(91.7)	Chinese Herbal Medicine	11(91.7)
			Acupuncture and Moxibustion	1(8.3)
			Therapeutic Massage	2(16.7)
			*Qigong*	1(8.3)
			Chinese Dietary Therapy	2(16.7)

Data are presented as the actual number of patients (percentage in that group).

Among the participants who used Chinese medicine (N = 449), 67.9% (N = 305) as recommended by relatives, ward-mates or others, while 52.1% (N = 234) made their own choice. Only 3.8% (N = 17) of the participants were recommended to use Chinese medicine by their physicians. Nearly two-thirds of the participants (N = 274) did not tell their physicians about using Chinese medicine. Of 175 participants who consulted their physicians about their use of Chinese medicine, 49.7% (N = 87) physicians were neutral, 29.1% (N = 51) were for, whereas 20.0% (N = 35) against their use of Chinese medicine (Table 5).

**Table 5:** Motivation for Chinese medicine use among patients and communication with Western medicine physicians

Characteristics	N (%)
Motivation for using Chinese medicine (N = 449)	
Recommendation from relatives, ward-mates or others	305 (67.9)
Will of patients themselves	234 (52.1)
Recommendations from physicians	17 (3.8)
Other	6 (1.3)
Consulted with Western medicine physicians about Chinese medicine use	
Yes	175 (39.0)
No	274 (61.0)
If 'yes', physician's response	
Encourage	51 (29.1)
Neutral	87 (49.7)
Discourage	35 (20.0)
Other	2 (1.1)
If 'no', why	
Doctor never asked	129 (47.1)
Patients thought Western medicine physician would not agree	116 (42.3)
Unnecessary to inform Western medicine physician	77 (28.1)
Other	9 (3.3)

Data are presented as the actual number of patients (percentage in that group).

### Factors contributing to treatment modality preference

With the hopes to reduce side-effects from Western medicine (65.5%), suppress tumor progression (60.8%), relieve symptoms (57.5%) and improve quality of life (48.4%), 54.2% of the participants (N = 426) preferred combined Chinese medicine and Western medicine treatments. Out of 347 (44.1%) participants who used Western medicine only, 67.7% did so because they believed that Western medicine alone could suppress tumor progression (Table 6).

**Table 6:** Patients' perspectives on the effectiveness of integrative Chinese and Western medicine (N = 786)

Variables	Western medicine n(%)	Chinese medicine n(%)	Integrative Chinese and Western medicine n(%)
Total	347(44.15%)	13((1.65%)	426(54.2%)
Progression suppress	235(67.7%)	9(69.2%)	259(60.8%)
Relieve the symptoms	83(23.9%)	10(76.9%)	245(57.5%)
Reduce dosage of western medicine			64(15%)
Sufficient psychological support	103(29.7%)	3(23.1%)	159(37.3%)
Sufficient evidence	185(53.4%)	2(15.4%)	
Promote the quality of life	103(29.7%)	8(61.5%)	206(48.4%)
Western medicine fails to suppress the progression		7(53%)	109(25.6%)
Chinese medicine fails to suppress the progression			77(18.1%)

**Figure 2:**
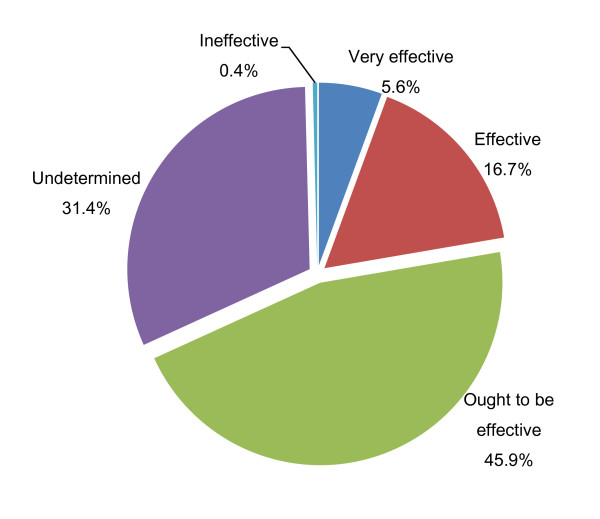
**Patients' intention in the use of Chinese medicine, Western medicine or integrative Chinese and Western treatment in the future (N = 786)**.

Over two-thirds of all participants (68.2%) believed that integrated Chinese and Western medicine was effective. Participants who were ambivalent about integrated Chinese and Western medicine effectiveness accounted for 31.4% (N = 245), while only 0.4% (N = 3) thought integrated medicine would not be effective (Figure 2).

**Figure 3:**
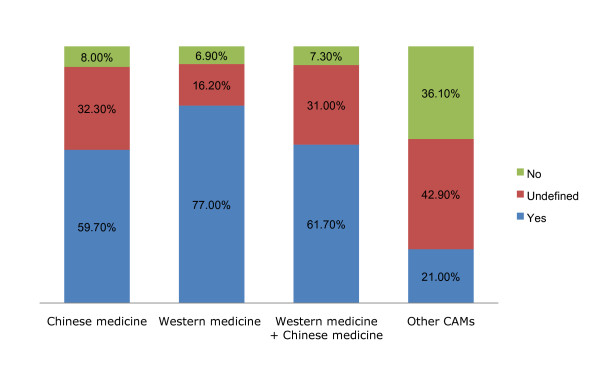
**Patients' satisfaction with their current treatment and the factors affecting their satisfaction levels**. Data are presented as the actual number of patients (percentage in that group).

A total of 469 participants (59.7%) claimed that they would continue or try Chinese medicine as an alternative therapy in the future, while 63 participants (8.0%) would not consider using Chinese medicine only (Figure 3).

### Demand for integrative Chinese-Western medicine treatment

Regarding whether or not the Hong-Kong government should further promote the integration of Chinese medicine and Western medicine in cancer treatment, 690 participants (87.8%) agreed, 92 (11.7%) had no opinion, while only 4 (0.5%) thought it was unnecessary.

## Discussion

Among many studies reporting the use of CAM to treat cancer patients [2-7,9,19-21], few reports were based on large-scale survey. This survey interviewed a large number of patients (N = 786) with various types of cancers to evaluate the characteristics of their treatment as well as their attitudes towards Chinese medicine treatment. The present study indicates that the use of Chinese medicine in cancer treatment in Hong Kong (57.1%) is much more than that in Japan (7.1%) [7], but lower than that in the mainland China (100%) [6]. As overseas Chinese often think of Chinese medicine as their first choice of CAM [22,23], this difference in the use of Chinese medicine may be due to socio-cultural difference among ethnics groups [22] rather than regional differences in medical systems.

Findings that cancer patients in Hong Kong favored Chinese herbal medication are consistent with previous studies [9,22]. The present study discovered that nearly half (49.93%) of the participants recruited from Western medicine clinics used Chinese medicine. Several factors were found to encourage the use of Chinese medicine among cancer patients [7,20,24,25], such as recommendations from the relatives and ward-mates, patients' own willingness, and advice from physicians.

Sixty-one per cent (61.0%) of the participants never talked to their physicians about their use of Chinese medicine. Half of the physicians (49.7%) held neutral opinions towards Chinese medicine use; one-third (29.1%) accepted Chinese medicine use and 20% rejected it. Seventy percent (70%) of the participants believed that integration of Chinese medicine and Western medicine would have positive effects in cancer treatment. Approximately 90% of all participants thought that the Hong Kong government should develop integrative Chinese and Western medicine in cancer treatment. As such, we propose that communication among patients, physicians and Chinese medicine practitioners should be encouraged.

Biases may exist in this study as a result of the non-randomized recruitment method. A total of 117 (14.9%) participants were recruited from three HKBU Chinese medicine clinics. The use of Chinese medicine among these participants may be higher than the participants from ordinary clinics. The recruitment from the Oncology Outpatient Department excluded those patients from the palliative day-care clinics. Nevertheless, among the participants (N = 669) recruited from Western medicine clinics, nearly half (49.93%) did use Chinese medicine. Even though the sample may not be representative of all cancer patients in Hong Kong, the large scale and interesting findings of this study does warrant a more structured and population-based sample in the future.

## Conclusion

Our findings indicate that most cancer patients in Hong Kong considered integrative Chinese and Western medicine as an effective cancer treatment. Randomized controlled trials to evaluate Chinese medicine treatments, establishment of integrative Chinese and Western medical facilities, and public education about Chinese medicine are greatly demanded.

## Competing interests

The authors declare that they have no competing interests.

## Authors' contributions

Bian ZX and Law CK conceived the study design, trained the research assistants, developed the study protocol and finalized the manuscript. Lam YC carried out the survey, performed data management and drafted the manuscript. Cheng CW checked the raw data and performed statistical analysis with assistance of Peng H. Huang XZ helped with participant recruitment. All authors read and approved the final version of the manuscript.
